# The use of personalized medicine for patient selection for renal transplantation: Physicians' views on the clinical and ethical implications

**DOI:** 10.1186/1472-6939-11-5

**Published:** 2010-04-09

**Authors:** Marianne Dion-Labrie, Marie-Chantal Fortin, Marie-Josée Hébert, Hubert Doucet

**Affiliations:** 1 Groupe de recherche en bioéthique, Université de Montréal, P.O. Box 6128, Downtown Station, Montreal, Quebec, H3C 3J7, Canada; 2 Centre Hospitalier de l'Université de Montréal (CHUM), Transplant Division, Hôpital Notre-Dame, 1560, Sherbrooke street East, Montreal, Quebec, H2L 4 M1, Canada; 3 Shire Chair in Nephrology and Renal Transplantation and Regeneration, Research Centre, Centre Hospitalier de l'Université de Montréal (CRCHUM), Hôpital Notre-Dame, 1560, Sherbrooke street East, Montreal, Quebec, H2L 2 M1, Canada; 4 Groupe de recherche en bioéthique, Université de Montréal, P.O. Box 6128, Downtown Station, Montreal, Quebec, H3C 3J7, Canada

## Abstract

**Background:**

The overwhelming scarcity of organs within renal transplantation forces researchers and transplantation teams to seek new ways to increase efficacy. One of the possibilities is the use of personalized medicine, an approach based on quantifiable and scientific factors that determine the global immunological risk of rejection for each patient. Although this approach can improve the efficacy of transplantations, it also poses a number of ethical questions.

**Methods:**

The qualitative research involved 22 semi-structured interviews with nephrologists involved in renal transplantation, with the goal of determining the professionals' views about calculating the global immunological risk and the attendant ethical issues.

**Results:**

The results demonstrate a general acceptance of this approach amongst the participants in the study. Knowledge of each patient's immunological risk could improve treatment and the post-graft follow-up. On the other hand, the possibility that patients might be excluded from transplantation poses a significant ethical issue. This approach is not seen as something entirely new, given the fact that medicine is increasingly scientific and evidence-based. Although renal transplantation incorporates scientific data, these physicians believe that there should always be a place for clinical judgment and the physician-patient relationship.

**Conclusions:**

The participants see the benefits of including the calculation of the global immunological risk within transplantation. Such data, being more precise and rigorous, could be of help in their clinical work. However, in spite of the use of such scientific data, a place must be retained for the clinical judgment that allows a physician to make decisions based on medical data, professional expertise and knowledge of the patient. To act in the best interests of the patient is key to whether the calculation of the global immunological risk is employed.

## Background

The present day overwhelming scarcity of organs for renal transplantation forces researchers, transplantation teams and national organizations responsible for allocation of organs to find new ways to resolve this issue. The growing reliance on living donors is one approach to easing the shortage [1], as is the establishment of new protocols, such as the removal of organs from a donor who has died after cardio-respiratory arrest [2], or the acceptance of altruistic donation [3,4]. There is another consequence of this organ scarcity: it encourages those involved in transplantation to make the best use of the resource. An ethical tension is thus created between two principles: promoting equity (giving everyone a chance to have access to transplantation), or maximizing efficacy (allocating organs to those recipients who would benefit the most). The criteria developed for the allocation of organs, which differ from one country to the other [5,6], attempt to reconcile this ethical tension. With the goal, always, of focusing on the best use of the resource, in order to increase the efficacy of renal transplantation recent scientific research is now focused on developing an approach based on personalized medicine.

Personalized medicine, arising from pharmacogenomics, is based on the premise: "the right treatment for the right patient at the right time" [7]. It attempts to direct the most appropriate medical interventions, choice of medications and preventive measures to a patient, related to quantifiable data from differing fields, by their genetic, clinical, psychosocial or other. This type of medicine even enables the specification of individual risks [8]. Most of the medical specialties, particularly oncology [9], are interested in personalized medicine. Transplantation medicine has recently joined them. Research has shown that their immunological and genetic characteristics predispose recipients to respond differently to immunosuppressive treatment after a graft [10,11]. For solid organs grafts, the precise and global identification of the genes and proteins involved in rejection enables specification of the potential immunological and genetic risks of rejection for each recipient, in addition to precise determination of the markers of survival for organs and grafts [12,13].

The Interdisciplinary Research Group on the Predictors of Immunological Risk, financed by the Fonds de la recherche en santé du Québec (FRSQ), is attempting to put in place a scientifically precise method for determining the global immunological risk (GIR) of rejection for each patient waiting for a renal transplant. This group of researchers has actually shown that the levels of genetic expression in the hematopoietic cells of the donor can predict the risk of graft versus host disease in bone marrow transplant cases [11]. Certain genes can thus be used to predict alloreactivity and rejection. In addition to these biological factors, others also play a role in the risk of rejection, particularly immunological, clinical and psychosocial factors [14,15]. Together, these factors (biological, immunological, clinical and psychosocial) could help to determine and to scientifically quantify the GIR of rejection for each potential recipient waiting for transplantation. This research methodology is in line with the approach of personalized medicine, since it facilitates identification of the best treatment for a patient, as well as the associated risks.

The precise determination of the GIR based on personalized medicine could effect huge changes to medical practice in renal transplantation. On the one hand, a personalized medicine approach could help to adapt the immunosuppressive therapy for each patient, and to predict more precisely each recipient's risk of rejection. Determination of the GIR could also help to improve treatment and follow-up, more accurately predict the outcome of a graft, and diminish the risks of organ rejection; this indeed is the goal of the research group.

On the other hand, evaluation of the GIR could also be used at the pre-graft stage to select recipients waiting for transplantation, even to play a role in the allocation of organs, revealing a utilitarian use of the GIR. This new method thus arouses numerous ethical questions. What are the benefits and the limits related to the use of this new method? How should decisions be made about its appropriate use? Does this approach constitute an entirely new approach? What are its impacts on clinical judgment and on the physician-patient relationship? What are the related ethical concerns? It is important to address these questions in concert with the perceptions that clinicians have concerning them, since they are the ones who will use the tools of personalized medicine.

The Interdisciplinary Research Group on the Predictors of Immunological Risk has thus carried out a qualitative research project focused on the predictors of immunological risk, in order to study the perceptions of those involved in renal transplantation (transplanting nephrologists and referring nephrologists) in the Province of Quebec regarding the use of the GIR based on a personalized medicine approach. This group has provided responses to the questions underlying this research, and has made possible a careful examination of the various perspectives of those involved in renal transplantation, from the viewpoint of scientific medicine.

## Methods

The participants chosen were nephrologists involved in patient selection and working in a renal transplantation centre in Quebec (transplanting nephrologists), or Quebec physicians specializing in nephrology and referring patients to a transplantation centre for a renal graft (referring nephrologists). The characteristics of the participants are summarized in Table 1. The research ethics committee of the University of Montreal has approved this research, and all of the participants have given their free and informed consent. Twenty-two semi-structured interviews were conducted with the participants between June 2007 and July 2008. The semi-structured interviews were used in order to discern the views of participants about the use within renal transplantation of the GIR based on personalized medicine. Each interview began with the presentation of short vignettes illustrating fictional clinical cases based on whether patients with specific medical conditions such as diabetes, the presence of genetic or immunological factors of rejection, would be accepted on the transplant waiting list. The vignettes were constructed based on discussions with transplant nephrologists and observation in a renal transplant team. One of these vignettes presented the definition of personalized medicine given to the participants: "Personalized medicine attempts to prescribe a therapy or an intervention for a specific patient, based on quantifiable information from a number of different domains, including the biological and the clinical. The use of the tools of personalized medicine could play a role in the assessment of patients for renal transplant by quantifying the different risk factors specific to these patients". The use of vignettes in qualitative research attempts to determine perceptions, attitudes and moral values, particularly pertinent for this study [16]. Open-ended questions were then directed to the participants. The number of semi-structured interviews was sufficient to attain the saturation and diversification of data [17], for both the transplanting and the referring nephrologists. The semi-structured interviews were all recorded; their average length was close to 60 minutes.

**Table 1:** Characteristics of the 22 participants in the research

Demographic characteristics	Respondents (n)	Respondents (%)
**Medical specialty**		
1. Transplant physicians (nephrologists)	12	54.55
2. Referring nephrologists	10	45.45

**Gender**		
1. Male	10	45.45
2. Female	12	54.55

**Milieus**		
1. Adult	19	86.36
2. Paediatric	3	13.64

**Age**		
1. 30-39	8	36.36
2. 40-49	2	9.09
3. 50-59	7	31.82
4. 60-69	5	22.73

**Years of practice**		
1. 0-9	8	36.36
2. 10-19	3	13.64
3. 20-29	6	27.27
4. 30-39	5	22.73

**Place of practice according to population**		
1. 0-99 000	2	9.09
2. 100 000-499 000	4	18.18
3. 500 000-999 000	2	9.09
4. 1 000 000-2 999 000	14	63.64

The transcription of the semi-structured interviews was qualitatively analyzed using the content and thematic analysis method described by Miles and Huberman [18]. A QSR N'Vivo (version 2.0) software was used to conduct a qualitative analysis in order to identify emergent themes, as well as the views of the participants. An independent researcher coded 10% of the raw data, and the rate of coding agreement was subsequently assessed (84%).

## Results

This section presents the different categories of the results: the views of participants about the GIR as a tool of personalized medicine, the benefits and limitations of such an approach, the perceived ethical issues associated with the GIR, and the perceived impacts on clinical judgment and the physician-patient relationship. Some of these themes, such as the benefits and limits of the approach, the impacts on clinical judgment and on the physician-patient relationship, clearly have an ethical component, but were not labeled as such by participants. The difference between these themes and the section on the perceived ethical issues, is that the latter is focused on physicians' answers to a specific question during the semi-structured interviews (ie. their views about what, for them, are the important ethical issues to consider when using the GIR in patient selection within renal transplantation).

### Views of participants about GIR as a tool of personalized medicine

The GIR based on personalized medicine is primarily seen as a tool to assist clinicians in their work. This approach is also seen as being objective, quantifiable and scientific; however the participants are very skeptical about attaining a high degree of precision in the evaluation of a patient's risk of rejection, in spite of the method's precision.

"I don't believe that we would ever have criteria that would discriminate well enough to be useful. This would give a low percentage: 1, 2, 3, 4, 5, 6%" (P19).

Regarding the clinical use of the GIR, a majority of the participants are in favour, as is shown in Table 2. However several participants mention more negative aspects, such as the difficulty of applying the method in the clinic, and the difficulty of accurately predicting the risk of rejection.

**Table 2:** Views of the participants about the use of GIR in renal transplantation

Perceptions	Respondents (n)	Respondents (%)
Favorable	**22**	**100.00**

Unfavorable	**14**	**63.64**

Neutral	**3**	**13.63**

In general, all of the participants are favorable to the calculation of the GIR, although 14 mention both neutral and favorable views, while 3 mention both favorable and negative views.

"There could be no objective method that would, with 100% accuracy, predict what is going to happen. There is no single objective method that could take into consideration all the characteristics" (P21).

The GIR is determined in relation to quantifiable factors: biological, immunological, clinical and psychosocial. Close to 68% of the participants mention that the factors must be weighted, and that they don't all have the same influence, as is shown in Table 3. Also, the factors must be determined in two ways: either in relation to the patient's situation or in relation to scientific models. The participants have not been explicit about ordering the importance of the factors, however they do give preference to biological, clinical and immunological factors based on objective data that can be verified using medical or scientific tests. The psychosocial factor is slightly different, but just as important as the other three, since it also contributes to the success of the graft [12,13]. It must be included in the GIR, especially if it helps to determine patient non-compliance, in spite of the difficulty of quantifying this more subjective factor.

**Table 3:** Weighting of the 4 factors involved in calculating the GIR

Weighting of the factors	Respondents (n)	Respondents (%)
Data are equal	**8**	**36.36**

Data are not equal	**15**	**68.18**

Depends on the situation, the context	**6**	**27.27**

Depends on scientific factors	**7**	**31.82**

"Every factor is different. You can't say: each is worth 10 points. It doesn't work that way. The refusal to take pills can't be compared with having a risk of rejection" (P6).

"It is certain that for any given patient, one of these factors will generally have more importance than the others" (P15).

"So if the older predictive models have already shown us that, for example, the psychosocial factors are less important than the immunological factors, that is good - I would weight them similarly" (P9).

The novelty of the method is an important element in the participants' views about the GIR. The use of a personalized medicine approach in transplantation should not be regarded as something entirely new. The criteria mentioned in the GIR are already used in transplantation, without precise weighing. The use of personalized medicine is viewed as an extension of the existing practice of renal transplantation.

"Establishing the risk is an exercise that is carried out with each patient. So there is nothing new in this approach" (P5).

### Benefits and limitations of the GIR in renal transplantation

**Figure 1:**
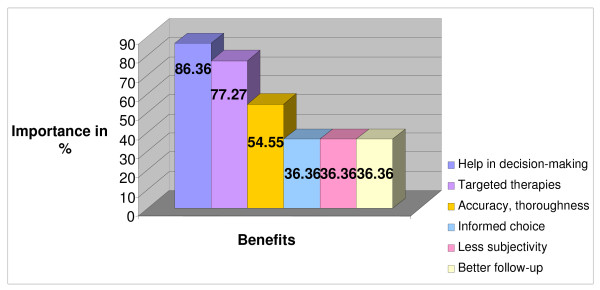
**Benefits of the GIR in Renal Transplantation**. Figure 1 indicates the main benefits, expressed in percentages, of the use of the GIR within renal transplantation mentioned by the participants. The percentage for each benefit corresponds to the number of times each of these was mentioned by the participants.

**Figure 2:**
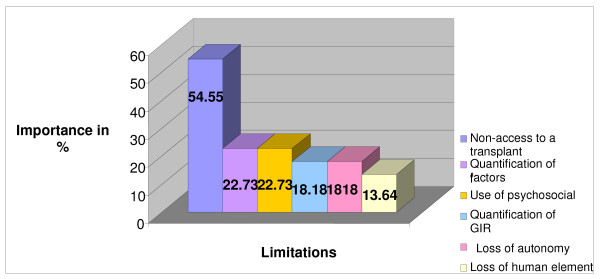
**Limitations of the GIR in Renal Transplantation**. Figure 2 presents the main limitations, expressed in percentages, of the use of the GIR within renal transplantation mentioned by the participants. The percentage for each limitation corresponds to the number of times each of these was mentioned by the participants. The main limitation is lack of access to a graft; followed by the difficulty of the risk associated with each of the 4 factors; the difficulty of quantifying criteria considered more subjective (psychosocial and clinical), particularly for specific patients; the difficulty of calculating the global risk; as well as physician loss of autonomy through use of the GIR, and the loss of the human side with this approach.

Figures 1 and 2 present the principle benefits and limitations of the use of the GIR. The principle advantage is the assistance that this approach gives to physicians in their decision-making, by consolidating their impressions and supporting their decisions. This is followed by the possibility of offering a therapeutic goal for each patient, and by the precision as well as the accuracy of the method. The possibility that patients will make a more informed choice, the assurance of a better follow-up, and a greater objectivity are also important advantages.

"I think that this will help us to make decisions which sometimes, in the absence of data, are based on expertise, or experience, or on 'gut feeling"' (P4).

"So if there are circumstances that lead me to suggest that there is a greater chance of rejection, certainly the follow-up and the treatment will perhaps not be the same as with someone whose risk is low" (P14).

"The advantage of this method is the attempt to bring a little more precision, clarity and accuracy" (P5).

The GIR also has its limitations, and raises concerns. The main limitation is the possibility that patients will not be accepted for a graft if their GIR is too high.

"But if one uses the data to say: 'this person can be grafted, the other cannot' - this, I believe is a problem, unless the factors reveal that the graft will definitely fail" (P15).

There are other difficulties associated with this approach: the difficulty of quantifying each of the factors, particularly the psychosocial and the global risk; the physician's loss of autonomy in whether or not to accept a patient on the waiting list for transplantation; loss of the human aspects of the physician-patient relationship and of the total picture of the patient.

"The risk is difficult to quantify. I don't think that we will ever be able to quantify a risk so precisely that we would be able to foresee perfectly the results of a transplantation" (P5).

"There is also the question of what takes place in your conversation with the patient. There is also a place for feeling. But the more precise the tool, the more one loses in these areas, while gaining in objectivity" (P11).

### The perceived ethical issues associated with the GIR

The participants point out several ethical concerns associated with the approach based on personalized medicine. Equity in the access to transplantation is an important ethical issue. A balance must be attained between efficacy and equity in the access to a graft within a context of scarcity. A second important ethical issue is the possible exclusion of patients because their risk of rejection is too high. The GIR can be used to help patients have access to transplantation by working on their risk, but it could also be used to select patients for renal transplantation in relation to whether their GIR is low or high.

"It is certain that with these tools, perhaps 98% of those grafted will have 10 good years with their graft. But perhaps we would have grafted only 75% of those we normally graft" (P7).

The participants mention as well that the establishment of an entirely new medical technology must be done in the interests of the patient. The establishment of this approach in the clinic must be very carefully evaluated, notably in relation to its impact on patients.

"I would be interested to see how things would change after 6 months to one year of use. Will this have changed the decision-making and what will be the repercussions for the patient? Will there be a suicide due to discouragement related to being taken off the list, or on the other hand, will there be gratitude? This is what I would look for: to be sure that this helps people rather than harming them" (P6).

Another ethical issue is who (physicians, patients, ethicists or society) should decide to establish personalized medicine in the clinic. The role of the physician should be minor in the taking of this decision. The participants grant a rather large place in this situation to the ethicist, who is recognized, according to one participant, as the moral authority for contemporary medicine.

"I think that the physician's place in this discussion is to present the data. What use is made of it, in my view, is not up to the physician" (P10).

### The perceived impacts on clinical judgment

Table 4 clearly indicates that, according to the respondents, clinical judgment will continue to play an important role in medicine, in spite of the increasing introduction of scientific data. This clinical judgment can be modified by the data which support the judgment of the clinician. Furthermore, close to 65% of the respondents mention that in the event of a discrepancy between the scientific data provided by personalized medicine and clinical judgment, the latter must take precedence.

**Table 4:** Displacement of clinical judgment by the GIR based on personalized medicine

Displacement of the clinical judgment	Respondents (n)	Respondents (%)
Personalized medicine will displace clinical judgment	**1**	**4.55**

Personalized medicine will not displace clinical judgment	**21**	**95.45**

"It is absolutely necessary to modify the latter, because when I look back just on the developments I have experienced, lets say for 30 years, there are things I do today that were not done earlier, because the means are available, from the immunological point of view, that were not available earlier" (P8).

"I imagine that my judgment will take the data into account! I should hold to my own judgment" (P17).

### The perceived impacts on the physician-patient relationship

The participants are divided on the question of whether the introduction of a personalized medicine approach is going to change the physician-patient relationship. Half of the participants think that there would be no change, as Table 5 indicates. One third of the participants think that the physician-patient relationship will change, particularly with the introduction of scientific data. The changes could be both positive (more informed choice for the patient, assistance in the decision-making for the physician), or negative (diminishing the role and the autonomy of the physician, patient resentment of the physician in the event of exclusion). Finally, about 13% of the participants believe that any change in the physician-patient relationship depends on the physician, on the patient, and on what uses are permitted for the GIR.

**Table 5:** Changes in the physician-patient relationship related to the use of the GIR

Changes in the physician-patient relationship related to personalized medicine	Respondents (n)	Respondents (%)
Yes, the relationship will change	**7**	**33.33**

No, the relationship will not change	**11**	**52.38**

It will depend on the physician, the patients and the use made of the approach based on personalized medicine	**3**	**14.29**

No response	**1**	**4.55**

"I think that if the relationship is basically good, it will not matter whether it is a scale that tells me that a patient is at risk, or whether it is my own opinion" (P14).

"Some patients always want to have more, and to know more. They will probably appreciate having access to data that is perhaps less "up in the air," while other patients will not be at all interested" (P4).

A majority of the participants mention that using the GIR would make it easier to tell patients that they cannot be grafted. This approach helps to justify a refusal to graft.

"There are going to be more elements with which to deal. But I want to say that it is never easy to refuse patients. But perhaps our decision-making is going to be easier, since we will have more information" (P20).

## Discussion

### Views on GIR as a tool of personalized medicine

The participants are favorable to the use of the GIR as a tool of personalized medicine, based on quantifiable factors, particularly given the goal of adapting post-graft treatment and working on patients' risks so that they would have access to a renal transplantation. With this approach, the more objective factors, such as the biological and the immunological, are considered to be the most important, even though markers for the latter are already used to match an organ with a recipient [19]. According to the respondents, the clinical factor is mainly considered to be an objective factor, based on medical tests that evaluate the patient's overall health status. It can also be seen as straddling the objective and the subjective. In fact, with a view to a graft, a patient's health status is evaluated by a transplanting nephrologist; and the impressions of the patient's physician also play a role in the clinical evaluation.

However, regarding the clinical situations presented in the vignettes, it was difficult for the participants to visualize the GIR's calculation and grouping together of the four quantifiable factors, so contrary to the current method which tends to separate the clinical and the psychosocial. The participants have a definite tendency to separate the data in the new method. They view them as quite unrelated, rather than imagining the data combined in order to calculate a global risk of rejection. What is important for them is obtaining precise data for each of the factors of individual risk.

According to some participants, only the biological criteria are new in this approach.

The possibility of the GIR being used to deprive patients of a renal graft poses a problem, unless there is a 100% chance of rejection. The goal of the pre-transplant evaluation is to improve the patient's quality and duration of life through transplantation and not to refuse access to a graft. Thus the participants question such use, especially if the patient realizes that while the use of the GIR has meant his being refused for renal transplantation, he would have been accepted by the current method. Refusing transplantation, based on genetic factors, also arouses concerns. These predictors of rejection do not depend on the willingness of the patients. Unlike psychosocial or clinical factors, neither the patient nor the medical team can do anything about this type of risk. The patient is not responsible for having genetic markers for rejection. This possible exclusion related to taking biological factors into account can also be seen as genetic discrimination in access to health resources, a theme which is not highlighted in this research, since legal protection against such discrimination already exists [20].

### Novelty of the method, importance of clinical judgment and the physician-patient relationship

The respondents do not believe that personalized medicine is such a novelty. It fits into the continuum of Western scientific medicine initiated by Francis Bacon (1560-1626). Bacon believed that science is a useful tool that enables modern medicine to prolong life and cure illness [21]. The introduction of scientific and objective data in medicine is in line with this ideal of an effective and efficient medicine seeking above all to relieve patients' suffering [22,23]. Moreover, the clinicians perceive that medical practice in renal transplantation is often personalized already. At the present time, a patient's medical evaluation for a possible renal transplantation seeks to identify the patient's risks of rejection based on clinical, immunological and psychosocial data [24,25]. On the other hand, the data is not quantified as it is in the approach based on personalized medicine.

It is also interesting to note t note note hat, for the participants, in spite of the inclusion of scientific and quantifiable data within medicine, clinical judgment remains important. It is humans who judge and assess the data and make final decisions about the treatment of patients, taking into account their knowledge of the patients. According to the respondents, no computer could achieve such an evaluation, based as it is on both objective and subjective factors. Clinical judgment essentially entails experience, intuition, feeling, and knowledge of the patient; it plays a role in the interpretation of data. Clinical judgment betokens a practical wisdom stemming from training and professional practice [26]. This judgment is involved in the relationship between the patient and the physician, as is dialogue, a communication between two players who seek the patient's good [27,28]. The participants believe that trust between the physician and the patient is the foundation of this relationship, which is complex in transplantation, since there are so many healthcare personnel involved with the patient (several specialists, psychologist or social worker, the transplantation team that decides whether or not a patient should be accepted for a transplantation, etc.). The addition of scientific data might introduce changes, but the relational aspect remains. Precise and thorough data will be given to the doctor and the patient, so that a shared decision can be taken. If clinical judgment retains its importance in this area, physician autonomy will not be diminished by the reference to scientific data. Physician interpretation of a patient's medical situation is crucial. More science within medicine does not necessarily lead to a loss of physician autonomy, deterioration of the physician-patient relationship or the clinical judgment.

### Importance of the principle of beneficence in the use of GIR

In their responses, the participants also highlight, sometimes implicitly, that the principle of beneficence is at the heart of their judgment. A physician must act in the best interests of the patient, and contribute to his/her wellbeing, as the Hippocratic Oath states: "I will apply dietetic measures for the benefit of the sick according to my ability and judgment; I will keep them from harm and injustice" [29]. In discovering and working on a patient's own risks, in order to facilitate access to renal transplantation, a clinician acts in the interests of the patient. On the other hand, if the data indicate that rejection is virtually assured, the physician would recognize that this must be taken into account, so that the patient's life would not be unnecessarily put at risk, particularly since dialysis, an alternate treatment, is available.

According to the participants, acting in the best interests of the patient tends to bring together two schools of thought usually seen as opposed: patient-centered medicine and scientific medicine [30]. Patient-centered medicine is based on the claim that patients must be active participants in their care [31,32]; while scientific medicine, mainly represented by the evidence-based paradigm, aims to base medical decision making about the care of an individual patient on the best possible evidence provided by scientific research [33]. In fact, as was noted above, the GIR must be used in the best interests of the patient. This implies that the participants, in order to build good relationships, must take into consideration the patient's story and his/her preferences; but it also implies that only medical treatment or intervention based on the best scientific information available will be offered. Patient-centered medicine and scientific medicine are thus intrinsically linked in the use of the GIR within renal transplantation.

### Who decides about the use of GIR?

An important question arises in relation to how personalized medicine is used: who must make decisions about its use? This question was a vital ethical issue for the participants. They did not feel that the physicians should decide on their own to use personalized medicine; the decision was one for society to make, based on its values and its priorities for transplantation. This view can be explained by the necessary involvement of the members of a society in instituting a system for allocating organs. Such a system is based on gift and on solidarity. If no one in society is willing to donate organs, whether during life or after death, the field of organ transplantation could not exist. Given such essential involvement, society has a right to make decisions about the graft of organs. However, several referring nephrologists also mention that the choice of whether or not to use the GIR must be left to the patients. Moreover, in accordance with the ideal of efficient medical practice, once a decision has been made to use this approach, it must be in line with the best scientific data on the subject.

It is interesting to note the crucial role in this that the participants assign to ethicists. As mentioned in the results, ethicists are viewed as being the moral arbiters within medicine. They encourage ethical reflection, and assist in resolving problematic situations, but are also perceived as those who suggest the most appropriate behavior, and the best course of action to follow. They are granted great authority in decision-making. Although the context is different, the respondents' position can be illustrated by the view of John Evans in his book, *Playing God*, focused on genetic engineering. Evans indicates how scientists have made bioethicists the experts for controversial questions in the biomedical sciences [34].

The participants' perception of the role of the ethicist can be questioned. Is the ethicist the initiator of moral norms? Can he/she impose behavior and alone reflect on the ethical problems linked to scientific development? In a sense, the respondents would like their choice of whether or not to use personalized medicine to be in line with "aiming at the good life with and for others in just institutions" [35]. In order to carry this out, they give a central role to the ethicist. Nevertheless, the establishment of moral norms, guidelines, and opinions is above all society's responsibility, and not that of the ethicist. From the beginning in dialysis, members of the community were involved in choosing the criteria for patient selection [36]. When new policies were created for organ allocation, far-reaching societal consultations usually took place [37,38]. Such choices are thus political. If the ethicist has a role to play, it would appear to us to differ from the one mentioned by the participants. The ethicist must be seen more as a catalyst who enriches the ethical reflection and promotes public discussion. So the role differs from that played when confronted by a specific case, when recommendations can be offered about "what to do in order to do the right thing?" On the other hand, as soon as political issues that concern the whole populace arise, the ethicist cannot act alone. In order to come to an ethical and political consensus, everyone's involvement must be promoted (citizens, politicians, those involved in the healthcare field).

### Balancing equity/efficacy in renal transplantation and the use of GIR

Another important ethical issue is finding a fair balance between equity, which means providing access to renal transplantation to those needing the resource, and efficacy [39]. In fact, seeking to improve the efficacy of the graft can contribute to limiting access, thus lessening equity, since fewer patients would be grafted. The method based on calculating the GIR could thus easily correspond to a purely utilitarian view of transplantation, which the participants reject. In their view, this method must not involve giving organs only to those who have no risk of rejection. To improve the efficacy of a graft does not necessarily imply a diminishing of equity. A personalized medicine approach is used in the interests of the patient. Determining a patient's GIR must enable the medical team to work to diminish this risk so that the patient would benefit from transplantation and so that the graft would last as long as possible. In this way, equity would not be affected by efficacy, and might even be improved. Some participants suggest that another way of viewing the equity/efficacy dilemma is to see it as the opposition between individual and collective rights. As in all other areas of health care, access to transplantation could be seen as a right [40]. In improving efficacy, the individual's right to access to a graft could be threatened if the GIR is used to select patients at low risk, rather than grafting all patients and then adjusting the immunosuppressive therapy. On the other hand, collective rights can be more carefully addressed by assuring a better efficacy for transplantations and better organ management. These remarks indicate a change in our view of transplantation: it has become a right, no longer a privilege [36]. The flourishing of individualism and civil rights that characterized the 1960's could explain this change of terminology and of vision.

## Conclusions

Is personalized medicine a way of the future in renal transplantation? The participants in this research believe that it is, and a majority of them are in favour of it, in spite of no consensus on the uses of this new approach. This method, viewed as a tool, is thought to be thorough, quantifiable and scientific. However, the participants do not view it as a novelty, but as part of a continuum designed to make medicine more scientific, efficacious and efficient. This approach improves physician's decision making, and adaptation of the therapy for each patient. On the other hand, there are doubts about how precisely the GIR can be quantified with this approach. Its principle limitations are the possibility of excluding patients, as well as the difficulty of quantifying the global risk and the factors considered more subjective (psychosocial). The factors that are quantified with this approach must not be seen as equal, and they must be weighted in relation to the patient's situation or scientific models.

The development of personalized medicine poses great challenges to clinical practice in renal transplantation. An ethical reflection is essential regarding the method's possible exclusion of patients, and on the delicate balance that must be established between the efficacy of transplantation and equity, within a context of scarcity.

The use of quantifiable data provided by personalized medicine appears to have little impact on clinical judgment and the relational aspect of the physician-patient relationship, which is founded upon trust. In spite of the growing reliance on scientific data, it is interesting to note that the participants in this research want to keep a place for clinical judgment. The interpretation of the data must be made by a physician, drawing on his/her own experience, judgment, medical expertise and knowledge of the patient. Clinical judgment must remain; it is a cornerstone of medical practice, which reconciles both the humanistic and the scientific aspects of medicine. In conclusion, research such as this is important in ensuring the appropriate development of personalized medicine within renal transplantation. This research clearly indicates the importance of empirical approaches in bioethics [41,42], which would provide assistance in dealing with the ethical issues identified by practitioners when personalized medicine is established in a clinical setting. It is also essential to examine patients' and society's views about the use of a personalized medicine method in kidney transplantation, by using an empirical approach. These views are currently under research within the Interdisciplinary Group on the Predictors of Immunological Risk.

## Competing interests

The authors declare that they have no competing interests.

## Authors' contributions

MDL was primary author of this paper. MCF participated in the inter-coding of the data, and has revised this article. MJH contributed to the editing and the revision of this article. HD contributed to the editing and the revision of this article. This article is a section of the doctoral thesis of MDL, of which MJH is the co-director and HD is the director. All authors have been directly involved in the writing of the final manuscript.

## Pre-publication history

The pre-publication history for this paper can be accessed here:

http://www.biomedcentral.com/1472-6939/11/5/prepub

## References

[B1] Baid-Agrawal S Frei UA Living donor renal transplantation: recent developments and perspectives Nat Clin Pract Nephrol 2007 3 31 41 10.1038/ncpneph0383 17183260

[B2] Kootstra G van Heurn E Non-heartbeating donation of kidneys for transplantation Nat Clin Pract Nephrol 2007 3 154 163 10.1038/ncpneph0426 17322927

[B3] Rodrigue JR Pavlakis M Danovitch GM Johnson SR Karp SJ Khwaja K Hanto DW Mandelbrot DA Evaluation of living Kidney Donors: Relationship types, Psyschosocial Criteria, and Consent Processes at US Transplant Programs Am J Transplant 2007 7 2326 2332 10.1111/j.1600-6143.2007.01921.x 17845566

[B4] BC Transplant Society performs Canada's first Living Anonymous Donor Transplant http://www.transplant.bc.ca/press_main.htm#firstLAD

[B5] Gillich M Heimbach D Schoeneich G Muller S Klehr H Comparison of blood group versus HLA-dependent transplantation and its influence on donor kidney survival Nephrol Dial Transplant 2002 17 884 886 10.1093/ndt/17.5.884 11981078

[B6] Koch T Normative and prescriptive criteria: the efficacy of organ transplantation allocation protocols Theor Med 1996 17 75 93 10.1007/BF00489742 8992648

[B7] Steele FR Personalized medicine: something old, something new Per Med 2009 6 1 5 10.2217/17410541.6.1.1 29783381

[B8] Burke W Psaty BM Personalized medicine in the era of genomics JAMA 2007 298 1682 1684 10.1001/jama.298.14.1682 17925520

[B9] Issa AM Personalized Medicine and the Practice of Medicine in the 21st Century McGill J Med 10 53 57 18523593 PMC2323540

[B10] Anglicheau D Legendre C Thervet E Pharmacogenetics in solid organ transplantation: Present knowledge and future perspectives Transplantation 2004 78 311 315 10.1097/01.TP.0000136256.56873.41 15316356

[B11] Baron C Somogyi R Greller LD Rineau V Wilkinson P Cho CR Cameron MJ Kelvin DJ Chagnon P Roy DC Prediction of graft-versus-host disease in humans by donor gene-expression profiling PLoS Med 2007 4 69 83 10.1371/journal.pmed.0040023 PMC1796639 17378698

[B12] Borozdenkova S Westbrook JA Patel V Wait R Bolad I Burke MM Use of proteomics to discover novel markers of cardiac allograft rejection J Proteome Res 2004 3 282 288 10.1021/pr034059r 15113105

[B13] Mansfield ES Sarwal MM Arraying the orchestration of allograft pathology Am J Transplant 2004 4 853 862 10.1111/j.1600-6143.2004.00458.x 15147418

[B14] Achille MA Ouellette A Fournier S Vachon M Hebert MJ Impact of stress, distress and feelings of indebtedness on adherence to immunosuppressants following kidney transplantation Clin Transplant 2006 20 301 306 10.1111/j.1399-0012.2005.00478.x 16824145

[B15] Fischer MS Psychosocial Evaluation Interview Protocol for Pretransplant Kidney Recipients Health Soc Work 2006 31 137 144 16776031 10.1093/hsw/31.2.137

[B16] Miles MB New methods for qualitative data collection and analysis: vignettes and pre-structured cases Int Qual Stud Educ 1990 3 37 51 10.1080/0951839900030104

[B17] Pires AP Poupart J, Groulx LH, Deslauriers JP, Laperrière A, Mayer R, Pires AP Échantillonnage et recherche qualitative: essai théorique et méthodologique La recherche qualitative: enjeux épistémologiques et méthodologiques 1997 Boucherville: GaëtanMorin 113 169

[B18] Miles MB Huberman MA Qualitative data analysis: An expanded sourcebook 1994 2 Thousand Oaks: Sage Publications

[B19] Martins L Fonseca I Sousa S Matos C Santos J Dias L Henriques A Sarmento A Cabrita A The influence of HLA mismatches and immunosuppression on kidney graft survival: an analysis of more than 1300 patients Transplant Proc 2007 39 2489 2493 10.1016/j.transproceed.2007.07.033 17954156

[B20] An act to prohibit discrimination on the basis of genetic information with respect to health insurance and employment [Genetic Information Nondiscrimination Act of 2008] http://frwebgate.access.gpo.gov/cgi-bin/getdoc.cgi?dbname=110_cong_public_laws &docid=f:publ233.pdf

[B21] Bacon F Francis Bacon's New Atlantis 2005 Manchester: Manchester University Press

[B22] Paul NW Fangerau H Why should we bother? Ethical and social issues in individualized medicine Curr Drug Targets 2006 7 1721 1727 10.2174/138945006779025428 17168846

[B23] Cassel EJ The nature of suffering and the goals of medicine N Engl J Med 1982 306 639 645 7057823 10.1056/NEJM198203183061104

[B24] Mandelbrot DA Pavlakis M Danovitch GM Johnson SR Karp SJ Khwaja K Hanto DW RodGIRue JR The Medical Evaluation of Living Kidney Donors: A Survey of US Transplant Centers Am J Transplant 2007 7 2333 2343 10.1111/j.1600-6143.2007.01932.x 17845567

[B25] Knoll G Cockfield S Blydt-Hansen T Baran D Kiberd B Landsberg D Rush D Cole E Canadian Society of Transplantation consensus guidelines on eligibility for kidney transplantation Can Med Assoc J 2005 173 S1 S25 10.1503/cmaj.1041588 PMC1330435 16275956

[B26] Ricoeur P Kemp P, Rendtorff J, Johansen NM Prudential judgment, deontological judgment and reflexive judgment in medical ethics Bioethics and Biolaw Vol 1 Judgment of life 2000 Copenhagen: Rhodos International Science and Art Publishers & Centre for Ethics and Law

[B27] McLeod ME Doctor-patient relationship: Perspectives, needs, and communication Am J Gastroenterol 1998 93 676 680 10.1111/j.1572-0241.1998.676_a.x 9625108

[B28] Warren J The doctor-patient relationship: A postmodern perspective Hum Health Care Int 1997 13 61 64 14986608

[B29] Veatch RM Cross Cultural Perspectives in Medical Ethics 2000 2 Boston: Jones & Bartlett Publishers

[B30] Bensing J Bridging the gap: The separate world of evidence-based medicine and patient-centered medicine Patient Educ Couns 2000 39 17 25 10.1016/S0738-3991(99)00087-7 11013544

[B31] Reynolds A Patient-centered care Radiol Technol 2009 81 133 147 19901351

[B32] Stewart M Towards a global definition of patient centred care BMJ 2001 322 444 445 10.1136/bmj.322.7284.444 11222407 PMC1119673

[B33] Bluhm R From hierarchy to network-a richer view for evidence-based medicine Perspect Biol Med 2005 48 535 547 10.1353/pbm.2005.0082 16227665

[B34] Evans JH Playing God? Human genetic engineering and the rationalization of public bioethical debate 2002 Chicago: University Of Chicago Press 10.1162/152651602317533802 22494192

[B35] Ricoeur P Oneself as Another 1992 Chicago: The University of Chicago Press

[B36] Fox RC Swazey JP The Courage to Fail. A Social View of Organ Transplants and Dialysis. Revised ed. published by University of Chicago Press, 1978 2002 New Brunswick: Transaction Publishers

[B37] Houssin D Organ shortage: A public health crisis. What is the French state doing about it? Transplant Proc 1997 29 3197 3198 10.1016/S0041-1345(97)00868-3 9414676

[B38] United Network for Organ Sharing: What we do? Policy development http://www.unos.org/whatWeDo/policyManagement/policyDevelopment.asp

[B39] Courtney AE Maxwell AP The challenge of doing what is right in renal transplantation: Balancing equity and utility Nephron Clin Pract 2009 111 C62 C67 10.1159/000180121 19060499

[B40] Goldberg AM Simmerling M Frader JE Why nondocumented residents should have access to kidney transplantation: Arguments for lifting the federal ban on reimbursement Transplantation 2007 83 17 20 10.1097/01.tp.0000247795.41898.55 17220784

[B41] Alvarez A How rational should bioethics be? The value of empirical approaches Bioethics 2001 15 501 519 10.1111/1467-8519.00258 12061377

[B42] Borry P Schotsmans P Dierickx K The birth of the empirical turn in bioethics Bioethics 2005 19 49 71 10.1111/j.1467-8519.2005.00424.x 15812972

